# The influence of paternal preconception health on birth defects and head circumference: A scoping review

**DOI:** 10.1371/journal.pgph.0005953

**Published:** 2026-02-13

**Authors:** Cindy-Lee Dennis, Karen McQueen, Justine Dol, Alistair Dennis-Grantham, Daisy R. Singla, Aimable Nkurunziza, Jennifer Abbass-Dick, Catherine S. Birken, Shefaly Shorey

**Affiliations:** 1 Lawrence S. Bloomberg Faculty of Nursing, University of Toronto, Toronto, Canada; 2 Department of Psychiatry, Temerty Faculty of Medicine, University of Toronto, Toronto, Canada; 3 Lunenfeld-Tanenbaum Research Institute, Sinai Health, Toronto, Canada; 4 School of Nursing, Lakehead University, Thunder Bay, Canada; 5 IWK Health, Halifax, Canada; 6 Campbell Family Mental Health Research Institute, Centre for Addiction and Mental Health, Toronto, Canada; 7 School of Nursing, Nipissing University, North Bay, Canada; 8 Arthur Labatt Family School of Nursing, Faculty of Health Sciences, Western University, London, Canada; 9 Faculty of Health Sciences, Ontario Tech University, Oshawa, Canada; 10 The Hospital for Sick Children, Toronto, Ontario, Canada; 11 Department of Paediatrics, Temerty Faculty of Medicine, University of Toronto, Toronto, Canada; 12 Alice Lee Centre for Nursing Studies, Yong Loo Lin School of Medicine, National University of Singapore, Singapore; 13 Director Yeo Boon Kim Mind Centre, National University of Singapore, Singapore; 14 Editor-in-Chief, Midwifery (Elsevier), Exeter, England; PLOS: Public Library of Science, UNITED STATES OF AMERICA

## Abstract

While paternal environmental exposures and lifestyle factors can influence sperm epigenetic states and affect fetal development, this area of research remains relatively underexplored. This comprehensive scoping review aimed to identify, compile, and analyze the literature on paternal preconception health and its impact on fetal development, specifically related to congenital birth defects (CBDs) and head circumference. We conducted a scoping review following the Joanna Briggs Institute methodology and a published protocol. Five databases were searched for articles that included men in the preconception period and outcomes for CBDs and head circumference. Studies were published in English up to July 16, 2025. Two independent reviewers screened titles and abstracts and extracted data from eligible studies using Covidence. Forty-eight studies were included in the review. We identified several paternal factors associated with CBDs, including paternal physical health (metabolic syndrome, viral infections, cancer), smoking and alcohol use, and environmental exposures (solvents, metals, pesticides). Most medications were not associated with increased risks; however, metformin and diazepam were identified as potential risk factors for increased CBD risk. The limited studies on head circumference also suggest a potential relationship; however, the findings are not widely applicable due to the small number of included studies. We also identified important knowledge gaps and methodological limitations that require further research to advance this field. Our findings indicate that paternal preconception health and exposures—particularly paternal health, substance use, environmental factors, and certain medications—significantly influence offspring health outcomes, including congenital defects and infant head circumference. These findings highlight the need to expand preconception counselling and preventive strategies to explicitly include fathers, with targeted efforts to improve paternal health, eliminate tobacco and alcohol use, and reduce occupational and environmental exposures. Incorporating paternal health into preconception frameworks is essential to understanding mechanistic pathways, decreasing congenital risks, and developing precision strategies for improving reproductive and neonatal outcomes.

## Introduction

### Background

Congenital birth defects (CBDs), also known as congenital disabilities, anomalies, or malformations, are structural or functional abnormalities that develop during embryogenesis [1]. CBDs affect about 6% of live births globally, roughly 8 billion, impacting physical, cognitive, and social well-being [2]. They are a leading cause of fetal death, neonatal mortality, and death under age five, and are associated with significant morbidity, as some CBDs can cause lifelong disabilities [1]. The exact statistics may be underestimated because stillbirths and terminations due to fetal anomalies are often excluded from the global disease burden estimates [3].

It is widely believed that the etiology of CBDs is multifactorial due to complex interactions between genetic and environmental factors [1,4]. Known maternal risk factors include teratogens, such as viruses (e.g., rubella) and the medication use during pregnancy (e.g., isotretinoin); health conditions (e.g., diabetes, obesity); and environmental exposures (e.g., alcohol, chemicals, smoking) [4–6]. Other maternal risks include genetic factors and chromosomal abnormalities [1,7]. While numerous maternal risk factors have been identified, the exact etiology of many CBDs is unknown [8].

There are concerns that the rates of CBDs may increase, with the alarming rise of known risk factors such as obesity and diabetes [5], as well as new or emerging threats like the Zika (ZIKV) epidemic [9] and assisted reproductive technologies (ART) [10]. A recent large cohort study in Denmark, Finland, Norway, and Sweden evaluated 7,747,637 liveborn children, including 171,735 conceived through ART, to assess whether children conceived via ART have a higher risk of congenital heart defects [10]. They found an increased risk of major congenital heart defects (aOR 1.36; 95% CI: 1.31, 1.41) and severe congenital heart defects (aOR 1.30; 95% CI: 1.20, 1.42) among ART-conceived singletons and multiples compared to singletons from spontaneous conception. The ZIKV outbreak in Brazil has also raised concerns over the past decade due to its international spread and teratogenic effects [9]. A review of 21 systematic reviews on health outcomes associated with ZIKV infection identified microcephaly (head circumference less than the third percentile for gestational age and sex) as the most common health outcome linked to ZIKV [9]. An additional concern is the rising rates of maternal obesity, which is linked to multiple types of CBDs that may affect cardiovascular, renal, and neurologic systems [11]. As such, efforts are necessary to prevent and identify risk factors for CBDs to reduce adverse effects on individuals, families, and the healthcare system.

There is strong evidence that maternal health before and during pregnancy plays a vital role in fetal development, and improving maternal health could prevent many CBDs. Therefore, preconception care that focuses on optimizing women’s health before pregnancy is of primary importance. However, preconception health should not focus solely on the mother, as paternal preconception health also matters, given the father’s epigenetic contributions. Due to the paucity of research on this topic, further research into paternal health factors and their impact on perinatal outcomes, including CBDs, is essential.

Enhanced recognition of the male’s contribution to child health, through both direct and indirect pathways, is emerging [12]. There is evidence which suggests that paternal environmental exposures and lifestyle factors can influence the epigenetic state of sperm and significantly impact offspring development [13]. Associations among paternal preconception variables such as alcohol [14], medications [15,16], and paternal health (e.g., metabolic syndrome) [17,18] and CBDs have been identified. Likewise, specific paternal preconception environmental exposures have been associated with smaller head circumference [19], a marker for brain development and cognition in later life [20]. While several single papers demonstrate a potential increased risk with paternal preconception exposures, other studies have found no increased risk [21–23]. Thus, a synthesis of the relevant evidence is essential to guide practice.

### Review Objective

The specific aim of this review was to identify, consolidate, and analyze the literature on how paternal preconception health affects CBDs and head circumference. This review was part of the Healthy Life Trajectories Initiative (HeLTI) Canada (www.helticanada.ca) and a larger scoping review [24] that examined the impact of paternal preconception health factors on perinatal and early childhood outcomes. Due to the wide variation in outcomes, the results are presented separately.

## Methods

### Design

A scoping review was selected as it aims to provide an overview of existing research, identify knowledge gaps and summarize the research. This review followed the Preferred Reporting Items for Systematic Reviews and Meta-Analysis (PRISMA) Extension for Scoping Reviews (PRISMA-ScR) [25] and the Joanna Briggs Institute (JBI) [26] methodology for conducting the review. It also adheres to a published protocol [24].

### Search strategy

A three-step process was used to identify published studies. First, the search keywords were refined and specified, and a preliminary search was conducted in Web of Science to test previously determined key terms. Second, an experienced health science librarian was consulted to ensure the accuracy of the search keywords and the resulting search strategy (S1 Table). The final search strategy, including all identified keywords and index terms, was adapted for each included database and/or information source. Databases searched included MEDLINE All (Ovid), Embase (Elsevier), CINAHL Full Text (EBSCO), Scopus (Elsevier), and PsycINFO (EBSCO). The search was conducted in all databases up to July 16, 2025.

### Inclusion criteria

#### Types of participants.

The review included all studies (quantitative, qualitative, mixed methods) involving men in the preconception period who are identified as the contributing procreation partner of a child for which direct or indirect outcomes were reported. Studies that solely reported on maternal exposures or did not separate paternal and maternal data were excluded. Although no time limits were applied to the search, only studies published from 2013 onward were included to reflect current evidence. The year 2013 was chosen because it incorporated the past decade of research, during which the initial searches were conducted. Experimental studies evaluating intervention effectiveness were also excluded, along with reviews, letters to the editor, editorials, commentaries, conference abstracts, dissertations, books, book chapters, and grey literature.

#### Concept.

CBDs are broadly defined to encompass a diverse range of terms, including but not limited to defects, anomalies, malformations, and disabilities.

### Study selection

All identified citations from the search were uploaded to Covidence, and duplicates were removed through their automated process. After pilot work confirming inclusion/exclusion criteria, titles, abstracts, and full texts were screened by two reviewers, with disagreements resolved by a third reviewer or through discussion. Reasons for exclusion at the full-text stage are reported.

### Data extraction and synthesis

The data extracted from full-text articles included specific information, such as study design, sample size, study participants, methods, paternal exposures and measurements, and main results. Data extraction was piloted before full data were extracted by one reviewer and verified by another. Data were organized by outcomes and summarized based on paternal preconception health exposures, CBDs, and head circumference. The findings are presented in narrative form, accompanied by tables and figures where appropriate. No quality appraisal was undertaken, consistent with the scoping review methodology [27].

## Results

### Search results

**Fig 1:**
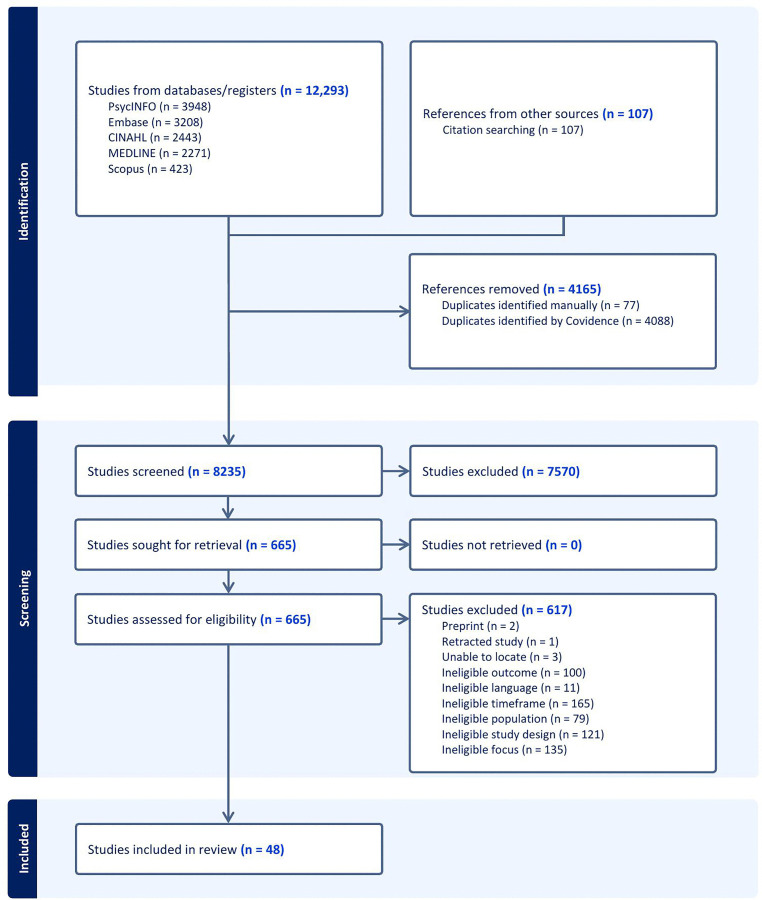
PRISMA Flow Diagram.

The systematic search found 12,293 citations across five databases. After removing duplicates, the titles and abstracts of 8,235 citations were examined. Full-text screening was conducted for 665 studies, with 48 meeting the inclusion criteria (Fig 1).

### Study and population characteristics

All 48 included studies were published from 2013 to 2025 (Tables 1 and 2). Most reported on CBDs (n = 42, 87.5%), while fewer examined head circumference (n = 6, 12.5%). The majority of studies took place in Denmark (n = 11, 22.9%), the United States (n = 11, 22.9%), and China (n = 9, 18.4%). Nearly all were cohort studies (n = 41, 85.4%). Sample sizes ranged from 58 to 8,787,096 fathers. However, in some large studies, not all male participants were exposed to the variable being evaluated (e.g., medication, smoking).

**Table 1:** Characteristics of Included Studies (Birth Defects).

Author/Year (Country)	Study Design (Sample)	Sample Size	Paternal Preconception Health Condition	Outcome	Congenital Birth Defect
**BMI**					
Chen et al., 2021 [28] (China)	Cohort (hospital-based)	5,741 fathers	BMI ≥ 25 kg/m2 or <25 kg/m2	Birth defect classified by ICD-10 codes (live births)	High BMI (≥25 kg/m2) was associated with higher risk of birth defects in IVF offspring.
Liu et et., 2025 [21] (China)	Cohort (population-based)	8,787,096 parent-child triads	BMI (kg/m2) Underweight (<18.5), healthy weight (18.5–23.9), overweight (24.0–27.9), and obesity (≥28.0)	Birth defects (any structural, functional, or metabolic abnormalities in the fetus before birth)	Paternal BMI (underweight, overweight and obesity) was not associated with a higher risk of offspring with a birth defect.
**Physical Health**					
Al-Jebari 2018 [29] (Sweden)	Cohort (population-based)	1,092,950 fathers	Cancers on the Swedish Cancer Register	Congenital malformation ICD-9/ICD-10 codes (live births)	Paternal cancer had a statistically significant increased risk of all malformations and major malformations.
Nie et al., 2020 [22] (China)	Case-control (population-based)	9,452 fathers	Disease (diabetes, fever, viral infection	CHD were coded by ICD-10 codes (live and stillborn fetuses)	Viral infection was associated with CHD. No association with fever or diabetes.
Yang et al., 2024 [30] (China)	Cohort (population-based)	6,675,540 fathers	HBV infection status (uninfected, previous infection, and new infection	Any CHD recorded on the birth defect registration card	Paternal previous HBV infection was independently associated with CHDs in offspring.
Yu et al., 2023 [18] (USA)	Cohort (national database, private insurance)	712,774 infants	Metabolic syndrome (MetS) and other chronic diseases	Structural birth defects identified by diagnosis code records (live births)	Association found between paternal MetS (2 + components) and birth defects.
Yu et al., 2023 [17] (USA)	Cohort (national database, private insurance)	376,362 male births	MetS components	Male genital malformations from ICD-10 codes (live births)	Two or more paternal MetS components increased the risk for the son being diagnosed with hypospadias.
**Smoking**					
Deng 2013 [32] (China)	Case-control (hospital-based)	653 infants fathers NR)	Smoking before pregnancy and through the first trimester	CHD diagnosed prenatally by echocardiography at greater than 14 weeks gestation	Paternal smoking was significantly associated with an increased risk of heart defects.
Nie 2020 [22] (China)	Case-control (population-based)	9,452 fathers	Smoking one or more cigarettes per day during the 3 months before pregnancy	CHD were coded by ICD-10 codes (live and stillborn fetuses)	Paternal smoking was associated with increased risk of congenital heart defects.
Ou 2015 [33] (China)	Case-control (population-based)	8,068 fathers	Smoking at least one cigarette per day	CHD diagnosis ICD-10 codes (live-born or stillborn fetuses over 17 weeks gestation)	Paternal smoking was associated with isolated CHDs.
Zhou 2020 (31) (China)	Cohort (population-based)	566,439 fathers	Preconception smoking	Birth defect diagnosis from hospital medical records in the first 42 days after delivery (all births)	Preconception paternal smoking increased the risk of congenital birth defects.
**Alcohol**					
Nie 2020 [22] (China)	Case-control (population-based)	9,452 fathers	Alcohol intake of at least 50 mL per day	CHD by ICD-10 codes (live and stillborn fetuses)	Paternal alcohol consumption was associated with an increased risk of CHDs.
Ou 2015 [33] (China)	Case-control (population-based)	8,068 fathers	Average of at least 50 ml per day, not specifying wine	CHD diagnosis ICD-10 codes (live-born or stillborn fetuses over 17 weeks gestation)	Paternal alcohol intake was associated with increased risk of CHDs.
Xia 2018 [34] (China)	Cohort (hospital-based)	592 infants (6 months)	Paternal exposure to alcohol at least once a week	Anogenital distance measured during a physical exam in the first 3 days, 6 months and 12 months (live births)	Paternal alcohol exposure was associated with shorter anogenital distance in boys and girls.
**Mental Health**					
Bukinowski et al., 2025 [35] (USA)	Cohort (national military data set)	19,149 infants born to 17,117 men	Post-traumatic stress disorder, major depression, panic/ anxiety disorder and any other mental health disorder (yes/no)	Major birth defects and ICD-9-CM diagnoses through the 1^st^ year of life (live births)	Men who screened positive for one or more mental health conditions versus none were not at significant risk for offspring with a birth defect.
**Environmental Exposures**					
Ali et al., 2014 [36] (Egypt)	Case-control (hospital-based)	240 fathers	Workplace exposure to hazards	Congenital malformations classified by ICD-10 codes (child presenting for surgery)	Exposure to pesticides, solvents, or welding fumes had higher odds of having a child with a congenital malformation.
Nie et al., 2020 [22] (China)	Case-control (population-based)	9,452 fathers	Environmental exposures	CHD were coded by ICD-10 codes (live and stillborn fetuses)	Exposure to organic solvents, living in a newly renovated room, traffic proximity, and paternal industrial occupation were positively associated with CHDs.
Tindula et al., 2021 [37] Bangladesh	Case-control (clinical)	278 fathers	Exposure to arsenic and 17 metals	Diagnosis of myelomeningocele or meningocele from a physician at a specified hospital (live infants < 1 year of age)	Exposure to arsenic, aluminum, cobalt, chromium, iron, selenium, and vanadium was positively associated with the risk of spina bifida.
Wu et al., 2022 [38] (China)	Case-control (hospital-based)	136 fathers	Hazardous substances	Treatment for anorectal malformation (live births)	Exposure to hazardous substances was significantly associated with anorectal malformations in univariate analysis, but this association was not significant in multivariate logistic analysis.
**Medication Use**					
Al-Jebari, 2019 [57] (Sweden)	Cohort (population-based)	1,167,665 fathers	Paternal anti-neoplastic treatment for testicular germ-cell cancer	Congenital malformation ICD-9 or ICD-10 (live births)	No additional risk of congenital malformation was identified with oncological treatment.
Boyer et al., 2020 [41] (France)	Cross-sectional (clinical)	1,332 fathers	Immunosuppressive agents for kidney transplant	Congenital anomaly self-report (live births)	No increased risk of congenital malformations when the father was exposed to immunosuppressive agents.
Eck et al., 2017 [44] (Denmark)	Cohort (clinical)	849,676 fathers	Methotrexate for systemic use	Congenital malformation classified by the European Surveillance System (live births)	No increased risk of major or minor congenital malformation when fathers were exposed to methotrexate.
Engeland et al., 2012 [61] (Norway)	Cohort (population-based)	349,020 fathers	Drugs dispensed 3 months before pregnancy.	Birth defects classified by ICD-10 (pregnancy length >12 weeks)	Birth defects were not increased when the father had been dispensed medications in any of the main anatomical therapeutic chemical groups, except for drugs for the nervous system, where increased odds of birth defects of the urinary system were identified.
Jones et al., 2013 (52) (USA)	Cohort (clinical)	152 fathers	Mycophenolic acid products among transplant recipients.	Malformations reported by the National Transplantation Pregnancy Registry or self-report (live births)	No specific patterns of malformation were identified. (similar to the general population).
Larsen et al., 2018 [40] (Denmark)	Cohort (population-based)	1,013,994 fathers	Corticosteroids within 3 months before conception.	Congenital anomaly was obtained by ICD-10 codes (live births)	No increased risk of congenital anomalies.
Larsen et al., 2016 [54] (Denmark)	Cohort (population-based)	399,870 fathers	Anti-TNF-α agents within three months of conception	Congenital anomaly data codes from ICD-8 or 10 (live births)	No increased risk of any congenital anomalies.
Lichtenstein, 2018 [55] (USA & Canada)	Cohort (clinical)	72 fathers	Infliximab for Crohn’s disease patients	Congenital anomaly at birth (live births)	No significant differences were observed among exposure groups for the proportion of congenital anomalies.
Lund et al., 2023 [39] (Denmark)	Cohort (population-based)	1,260,934 fathers	Non-steroidal anti-inflammatory drugs and opioids	MCMS were classified according to the EUROCAT guidelines using ICD-10 codes within 1 year after birth (live births)	No clinically important associations between paternal preconception exposure and major congenital malformations.
Madabhavi et al., 2019 [58] (India)	Cohort (clinical)	58 fathers	Imatinib and had chronic myeloid leukemia	Congenital malformation in full-term pregnancy (live births)	No increased risk of any congenital malformations.
Martin-Moreno et al., 2021 [53] (Spain)	Cohort (clinical)	151 fathers	Mycopholenic acid (MPA) and male kidney transplant	Birth defects self-report (live births)	The incidence of congenital birth defects in the exposed group (4%) was similar to other studies.
Meng et al., 2024 [23] (Norway and Taiwan)	Cohort (population-based)	Offspring 616,389 (Norway) 2,563,812 (Taiwan)	Metformin	Congenital anomaly according to the EUROCAT guidelines	Paternal preconception metformin use was not associated with birth defects in the adjusted analysis.
Meserve et al., 2021 [62] (USA)	Cohort (medical/ pharmacy claims)	7,453 fathers	Immunosuppressive or biologic agents	MCMs based on WHO criteria and identified as having at least 2 ICD-9/10 codes (live births)	There was no significant association between paternal exposure to any immunosuppressive or biologic medication and risk of major congenital malformations.
Morken et al., 2015 [42] Norway	Cohort (population-based)	2,463 fathers	Immunosuppressive agents	Congenital malformations using ICD 9–10 codes and EUROCAT guidelines	No increased risk was found for congenital malformations.
Nørgård et al., 2022 [48] (Denmark)	Cohort (nationwide)	9,900 fathers	5- aminosalicylic acid	Major congenital anomalies ICD-10 codes (live births)	No increased risk of major congenital anomalies.
Nørgård et al., 2022 [50] (Denmark)	Cohort (population-based)	7,648 fathers	Antidiabetic drugs three months prior to conception.	MCMs were identified in the Danish National Patient Registry, ICD-10 codes (live births)	Metformin exposure was associated with an increased risk of major congenital malformation. No association with insulin or sulfonylureas.
Nørgård et al., 2017 [49] (Denmark)	Cohort (nationwide)	1,013,323 fathers	Azathioprine and 6-mercaptopurine	Congenital anomalies ICD-10 (live births)	No significant increased risk of any congenital anomaly.
Uyaroglu, 2020 (56) (Turkey)	Cohort (clinical)	45 fathers	Anti-tumour necrosis factor alpha (anti-TNF) agents.	Congenital malformations (not defined) (live births)	No congenital malformations were found.
VanderMijle, 2023 [43] Netherlands	Cohort (population-based)	15,959 fathers	Long-term immunosuppressants	MCM from EUROCAT guidelines (live births)	Two (3.7%) major congenital malformations were reported. The data did not show an increased risk.
Viktorin et al., 2018 [51] (Sweden)	Cohort (population-based)	170,508 fathers	Fathers receiving antidepressant treatment	Any malformation according to ICD-10 codes (live births)	Paternal antidepressant use during conception was not associated with any malformations.
Wallenius et al., 2015 [60] (Norway)	Cohort (nationwide)	1,796 fathers	Disease-modifying anti-rheumatic drugs for inflammatory joint disease	Severe (major) malformations ICD-10 codes (live births and stillbirths after 12 weeks gestation	No increase in the risk of severe congenital malformations.
Weber-Schoendorfer et al., 2013 [45] (Germany)	Cohort (clinical)	525 fathers	Low-dose methotrexate	Major or minor birth defects classified by 2 of the authors based on standard two classification systems (live births, pregnancy losses and terminations)	The rate of birth defects did not differ significantly between groups.
Wensink, 2022 (16) (Denmark)	Cohort (population-based)	936,706 fathers	Paternal intake of neurologic drugs (e.g., antidepressants, sedatives)	Major birth defect by EUROCAT guidelines and ICD codes (stillbirths excluded)	Weak or null associations between birth major defects and the father’s intake of medications.
Wensink, 2022 (15) (Denmark)	Cohort (population-based)	Not reported	Diabetes drugs	Major birth defect by EUROCAT guidelines (stillbirths excluded)	Metformin exposure was associated with increased odds of having one or more birth defects. No increased odds for insulin or sulfonylurea.
Winter et al., 2017 [46] (Denmark)	Cohort (population-based)	1,013,994 fathers	Methotrexate within three months before conception	Congenital anomalies up to 1 year of age and ICD-10 codes (live births)	No increased odds of any congenital anomalies.
Wu et al., 2021 (38) (China)	Case-control (hospital-based)	136 fathers	Antibiotics, analgesics, nonsteroidal anti-inflammatories, acid inhibitors, antihypertensives, or antidiabetic drugs	Treatment for anorectal malformation (live births)	Preconception medication use was associated with anorectal malformation.
Yang et al., 2019 [59] (Denmark)	Cohort (population-based)	733,282 fathers	Anti-epileptic drugs three months before conception	Congenital anomaly based on ICD-10 codes (live births)	Anti-epileptic drug exposure was associated with an increased risk of congenital anomalies.
Zarén et al., 2023 (47) Sweden	Cohort (population-based)	809,929 fathers	Methotrexate	Congenital anomalies (major/minor) from ICD-10 codes and EUROCAT guidelines (live births)	No increased risk.

BMI: body mass index; CHD: congenital heart disease; EUROCAT: European Surveillance of Congenital Anomalies; HBV: hepatitis B virus; MCMs: major congenital malformations; MetS: metabolic syndrome

**Table 2:** Characteristics of Included Studies (Head Circumference).

Author/Year (Country)	Study Design	Sample Size	Paternal Health Condition	Head Circumference (HC)
**Alcohol**				
Zuccolo et al., 2016 [63] (Norway)	Cohort (secondary data)	46,178 trios	Paternal average alcohol dose per occasion	Paternal alcohol consumption (5 + units compared to none) had higher odds of an infant being born with microcephaly.
**Environmental Exposures**				
Bloom et al., 2015 (19) (USA)	Cohort (LIFE) (clinical)	501 couples	Presumed exposure to persistent organic pollutants	Higher paternal urine Uranium was associated with smaller HC. Higher molybdenum levels were also associated with smaller HC in boys.
Messerlian et al., 2018 [65] (USA)	Cohort (EARTH) (clinical)	184 fathers	Phenol exposure	No associations with paternal phenol exposure and HC.
Mustieles et al., 2018 [66] (USA)	Cohort (EARTH) (clinical)	190 fathers	Bisphenol exposure	No associations with paternal preconception urinary BPA concentrations and HC.
Robledo et al., 2015 [64] (USA)	Cohort (LIFE) (clinical)	234 fathers	(PBB, OCPs, PBDEs, PCBs, and PFCs)	Paternal PCBs were associated with smaller HC among girls (PCB-167) and boys (PCBs 128, 157).
Smarr et al., 2015 [67] (USA)	Cohort (clinical)	233 fathers	BPA, phthalates, and creatinine	No associations were observed between paternal urinary chemicals and HC

BPA: Bisphenol A; HC: head circumference; PBB: polybrominated biphenyl; OCP: organochlorine pesticides; PBDE: polychlorinated diphenyl ethers; PFC: perfluoralkyl chemicals; ICD: International Classification of Diseases transplant, irritable bowel disease, leukemia, inflammatory diseases, epilepsy, diabetes, and others.

### Data synthesis

Findings from this review first report on CBDs and then on head circumference based on paternal preconception health categories (as applicable): physical health, substance use, mental health, environmental exposures, and treatment effects.

## Congenital birth defects outcome

### Paternal physical well-being

***Body Mass Index (BMI).*** Two studies examined the association between paternal BMI and the risk of birth defects, yielding mixed results. A US cohort study evaluated paternal preconception BMI among 5,741 couples undergoing fertility treatment. They found that a higher paternal preconception BMI of ≥25 kg/m^2^ was associated with an increased risk of birth defects (aOR 1.82, 95% CI: 1.06, 3.10) in in vitro fertilization (IVF) offspring compared to those with lower BMI (<25 kg/m^2^) [28]. However, in the subgroup analysis, only the odds of congenital malformations of the musculoskeletal system were significantly higher in IVF offspring with a paternal BMI of ≥25 kg/m^2^ (aOR 4.55, 95% CI: 1.32, 15.71). Alternatively, a large population-based Chinese cohort study (n = 8,787,096) found that men in the preconception period who were underweight, overweight, or obese were not at a higher risk of having an offspring with a birth defect compared to those with a normal BMI [21].

***Physical health.*** Five studies examined the influence of paternal preconception physical health status on birth defects. These health conditions included cancers [29]; diabetes, fever, and viral infections [22]; hepatitis B virus [30]; and components of metabolic syndrome (MetS) [17,18]. Two large US cohort studies using data from the IBM Marketscan Research Database found that  fathers with preconception components of MetS had an increased risk of birth defects. In the first study (n = 712,774 live births), fathers with MetS prior to conception had a higher percentage of infants with cardiac birth defects compared to fathers without MetS (MetS = 1, aOR 1.07; 95% CI: 1.01, 1.13); (MetS = 2, aOR 1.17; 95% CI: 1.08, 1.26) [18]. Similarly, fathers with two or more MetS components (aOR 1.45; 95% CI: 1.22, 1.71) had a higher proportion of infants with respiratory defects than fathers without MetS. The second study, which evaluated male infants (n = 376,362), found that fathers with two or more MetS components had increased odds of a son being diagnosed with hypospadias (aOR 1.27; 95% CI: 1.10, 1.47) [17]. Another large cohort study conducted in Sweden assessed the associations between paternal cancer (diagnosis obtained from the Swedish Cancer Register) and congenital malformations in offspring [29]. They found that children conceived before paternal cancer had a statistically significant increased risk of all malformations (OR 1.08, 95% CI: 1.02, 1.15) and major malformations (OR 1.09; 95% CI: 1.01, 1.18), compared to those conceived after paternal cancer. Lastly, two studies evaluated preconception viral infections. An extensive retrospective cohort study in China (n = 6,675,540) found that prior paternal hepatitis B virus (HBV) infection was independently associated with congenital heart defects (CHD) in offspring (aRR 1.40; 95%CI: 1.11-1.76) [30]. Similarly, a case-control study conducted in China found that fathers with a preconception viral infection had a significant increase in CHDs among offspring (cOR 3.00; 95% CI: 1.94, 4.46) [22]. Two cardiologists reviewed all echocardiograms of CHD cases. No association was found between paternal fever or diabetes and the occurrence of birth defects.

### Paternal substance use

***Smoking.*** Four studies conducted in China with varied sample sizes (n = 653–566,439 couples) examined paternal preconception smoking and CBDs. The findings from the studies demonstrated a significant and direct correlation between preconception paternal smoking and increased birth defects. In an extensive population-based cohort study (n = 566,439), the preconception fathers who continued smoking (OR 1.87; 95%CI: 1.36, 2.56) or decreased smoking (OR 1.41; 95%CI: 1.10, 1.82) had an increased risk of birth defects (e.g., CHDs, limb abnormalities, and neural tube defects) compared with the fathers who did not smoke [31]. In the case-control analysis, infants whose fathers stopped (OR 0.32; 95% CI: 0.15, 0.67) or decreased their smoking (OR 0.25; 95% CI: 0.13, 0.49) were at lower risk than those whose fathers continued smoking [31]. Similarly, another case-control study of 653 fathers found that paternal preconception smoking that was light (aOR 2.23; 95% CI: 1.05, 4.73) to heavy (aOR 8.16; 95% CI: 1.13, 58.84) was associated with CHD among infants [32]. In particular, there was an increased risk of CHDs, septal defects, and left ventricular outflow tract obstructions. Two additional case-control studies identified that paternal smoking (n = 9,452 and n = 8,069) was associated with an increased risk of CHDs (OR 1.76; 95%CI: 1.54, 1.98) [22] and isolated CHDs (OR 1.76; 95%CI:1.40, 2.21) [33], respectively.

***Alcohol*.** Three studies examined paternal preconception alcohol exposure and identified a positive association between paternal preconception alcohol use and CHDs [22,33] and anogenital distance (AGD) [34]. A population-based case-control study conducted in China (n = 4,267 cases and controls) found paternal alcohol consumption was associated with an increased likelihood of CHDs (aOR 2.87; 95% CI: 2.25, 3.65 [22]. Another case-control study in China found paternal alcohol intake was associated with an increased risk of CHDs including atrial septal defects (OR 3.39; 95% CI: 1.96, 5.84), ventricular septal defects (OR 1.75; 95% CI: 1.16, 2.64), isolated CHDs (OR 2.14; 95% CI: 1.64, 2.80), and multiple defects (OR 4.99; 95% CI: 1.34, 18.52) [33]. Lastly, in a cohort study conducted in China, the AGD was measured for infants at birth (n = 980), at 6 months (n = 592, 60.4%), and 12 months (n = 543, 55.4%) as a sensitive marker of reproductive organ development and reproductive function [34]. Boys in the paternal alcohol-exposed group had shorter AGDs, regardless of the areas measured or the time of measurement, compared to those in the unexposed group. However, only the differences in AGD-anus to penis at birth and AGD-anus to scrotum at 6 months were statistically significant. For girls, the associations were similar at birth (shorter AGD among the alcohol-exposed group); however, at 12 months, the measurements were not significantly different between exposed and unexposed girls.

### Paternal mental health

***Mental health.*** Only one study examined paternal preconception mental health among military service men (n = 12,117) and major birth defects using secondary data from two databases [35]. Mental health exposures included post-traumatic stress disorder, major depression, and panic/anxiety disorder and were categorized as “any mental health condition” (yes or no). Having a mental health condition (yes/no) did not significantly increase the risk of birth defects among offspring, nor did having multiple mental health conditions (e.g., 2 or 3 conditions).

### Paternal environmental exposures

***Environmental exposures.*** Four studies identified consistent links between paternal preconception exposure to various environmental hazards and birth defects. A case-control study in Egypt (n = 240 fathers) examined the relationship between paternal workplace exposures and the risk of musculoskeletal congenital malformations [36]. They found that paternal preconception occupational exposure to pesticides (OR 3.40; 95% CI: 1.94, 5.88), solvents (OR 5.69; 95% CI: 2.88, 11.52), and welding fumes (OR 2.80; 95% CI: 1.19, 7.28) was associated with an increased likelihood of children with musculoskeletal congenital malformations. Another case-control study explored the effects of several environmental exposures on CHDs in a sample of 9,542 couples in China [22]. It found significant positive associations between paternal environmental exposure to organic solvents (OR 4.44; 95% CI: 2.18, 9.6), living in newly renovated rooms (OR 2.98; 95% CI: 2.08, 4.26), residing near a main road (OR 2.11; 95% CI: 1.77, 2.50), and having an industrial occupation (OR 1.95; 95% CI: 1.63, 2.34) with CHDs. In a Bangladeshi case-control study, researchers investigated the relationship between paternal exposure (n = 278) to various metals and the risk of neural tube defects in children via toenail samples from parents [37]. A one standard deviation increase in the natural log of paternal preconception exposure to arsenic was linked to a 74% higher likelihood of a neural tube defect in adjusted models (OR 1.74; 95% CI: 1.26-2.42). Adjusted models of paternal preconception exposure to aluminium (OR 1.42; 95% CI: 1.04–1.93), cobalt (OR 1.46; 95% CI: 1.07- 2.0), chromium (OR 1.43; 95% CI: 1.06-1.92), iron (OR 1.57; 95% CI: 1.17- 2.11), selenium (OR 12.51; 95% CI: 4.33- 36.18, P < 0.001), and vanadium (OR 1.40; 95% CI: 1.05-1.88) also showed a positive association with the risk of spina bifida in children. A case-control study in China involving 136 couples examined various paternal exposures to hazardous substances (X-rays, organic solvents, industrial waste gas, metals, and pesticides) and anorectal malformations in children [38]. Although a significant association was identified in univariate analysis (Crude OR 3.31; 95% CI: 1.63, 6.74), it did not persist in the logistic analysis (p = 0.11).

### Paternal medication treatment effects

***Medication use.*** Numerous studies (n = 28) assessed various paternal preconception medication use and the risk of birth defects. In most studies (n = 25 of 28, 89.3%), the risk of birth defects was not significantly higher among offspring exposed to the medication(s) during the preconception period. The types of medications before conception included non-steroidal anti-inflammatories and opioids [39], corticosteroids [40], immunosuppressants [41–43], methotrexate [44–47], 5-aminosalicylic acid [48], azathioprine (AZA) and 6-mercaptopurine (6-MP) [49], antidiabetic [15,23,50], nervous system [16,51], mycopholenic acid [52,53], anti-TNF [54–56], anti-neoplastic [57], imatinib [58], anti-epileptic [59], disease-modifying anti-rheumatic [60] and other diverse medications [38,61,62]. These medications were for various conditions such as organ

In three of the studies, preconception paternal medication exposure was associated with a higher risk of birth defects [15,38,50], and three studies showed mixed results [23,59,61]. In a Chinese study of 136 children with anorectal malformations, researchers found that paternal drug use six months prior to conception was associated with a higher risk of these malformations (aOR 9.17; 95% CI: 2.19, 38.49) [38]. Paternal drug use included preconception use of antibiotics, analgesics, nonsteroidal anti-inflammatory drugs, acid inhibitors, antihypertensives, or antidiabetic medications. In two Danish studies assessing anti-diabetic medications, an increased risk of birth defects was observed with paternal preconception use of metformin; however, no association was found with preconception use of insulin or sulfonylureas [15,50]. They noted that fathers’ use of metformin was linked to a higher risk of major congenital malformations (OR 1.40; 95% CI: 1.11, 1.76) [50]. Their findings suggested an additional 14 major congenital malformations per 1000 fathers exposed to metformin. Similarly, Wensink et al. [15] reported that offspring exposed to metformin (n = 1,451) had an increased rate of birth defects (aOR 1.40; 95% CI: 1.08, 1.82). They also found that among metformin-exposed offspring, genital defects in boys were common (aOR 3.39; 95% CI: 1.82, 6.30). However, a recent large cohort study in Norway (n = 616,389 offspring) and Taiwan (n = 2,563,812 offspring) did not support these findings. Meng et al. (2025) found no association between preconception paternal metformin use and congenital anomalies in the adjusted analysis, which was restricted to fathers with type 2 diabetes.

Two studies assessing paternal preconception treatment and birth defects yielded inconclusive results. In the study by Engeland and colleagues [61], researchers found that the odds of congenital anomalies were not increased for any drug within the main anatomical therapeutic chemical groups. However, subgroup analysis revealed that drugs for the nervous system (e.g., diazepam) were linked to a higher chance of birth defects of the urinary system (OR 1.5; 95% CI: 1.2, 2.0). In another study, researchers identified a greater risk of congenital anomalies among the offspring of fathers taking antiepileptic drugs [59]. Nonetheless, the studies also detected an increased risk of congenital anomalies at other times—beyond or after the 3-month preconception period—implying that paternal epilepsy may independently raise the risk of malformations rather than the medication treatment itself.

## Head circumference outcome

### Paternal substance use

***Alcohol***. One study in Norway investigated the association between paternal preconception alcohol and infant head circumference using data from the Norwegian Mother and Child Cohort Study (n = 68,244 mother-father-offspring trios) [63]. No association was found between preconception paternal alcohol intake and head circumference when modelled as a continuous outcome. However, they found some evidence suggesting higher preconception paternal alcohol consumption (5 + units versus none; OR 1.93; 95% CI: 1.01, 3.70) was associated with increased odds of being born with microcephaly.

### Paternal environmental exposures

***Environmental exposures***. Five studies examined the effect of paternal preconception exposure to organic pollutants and synthetic chemicals on infant head circumference, yielding mixed results. One study assessed exposure to persistent organic pollutants (POPs) among couples (n = 501) and their infants (n = 235) participating in the LIFE study in the US [19], finding that higher paternal urine concentrations of uranium were associated with smaller head circumferences (-0.83 cm [-1.60, -0.05]). Paternal urine levels of molybdenum were also associated with smaller head sizes in boys (-0.57 cm). In another US LIFE study [64], the relationship between serum concentrations of 63 POPs in couples (n = 234) and the head circumference of their singleton children (n = 234) was explored. Paternal concentrations of polychlorinated biphenyl (PCB) 167, as well as PCBs 128 and 157, were linked to reduced head sizes in both girls (β = –0.45 cm; 95% CI: –0.86, –0.03) and boys (β = –0.66 cm; 95% CI: –1.31, –0.01; β = –0.54 cm; 95% CI: –1.01, –0.06). Three cohort studies examined the association between exposure to synthetic substances and infant head size. The two US Environment and Reproductive Health (EARTH) studies assessed paternal exposure to phenols (e.g., benzophenone-3, triclosan, butylparaben, propylparaben, methylparaben, and ethylparaben) [65] and bisphenols (e.g., BPA and BPS) [66], with no findings linking these exposures to head size. Similarly, another US study reported no association between paternal exposure to bisphenol A, phthalates, creatinine, and head circumference [67].

## Discussion

This review is the first to comprehensively synthesize evidence on paternal preconception health and its impact on congenital birth defects (CBDs) and infant head size. Across studies, paternal health conditions (e.g., metabolic syndrome, viral infections, cancer), smoking, alcohol consumption, and occupational or environmental exposures (solvents, metals, pesticides) were associated with elevated risks of congenital anomalies, especially affecting cardiac, musculoskeletal, and neural tube development. Most paternal medications were not associated with higher risks, though some links were found with metformin and certain nervous system drugs. Evidence regarding paternal BMI was inconsistent, and only one study addressed paternal mental health, highlighting a significant research gap. Research on infant head size remains limited and inconclusive, though some studies suggest paternal alcohol use might increase microcephaly risk, while exposure to organic pollutants (uranium, molybdenum, PCBs) has been associated with smaller head circumference. Overall, these findings emphasise the vital role of paternal factors in shaping offspring health and highlight the importance of including paternal considerations in preconception care alongside maternal health.

### Congenital birth defects

The results indicate a significant influence of paternal health conditions, such as metabolic syndrome, cancer, or viral infections, on birth defects. This aligns with the study by Ståhl and colleagues [68] who reported an increased risk of major CBDs in the offspring of cancer survivor fathers, especially when conception occurred within two years of diagnosis. While paternal metabolic syndromes and viral infections during preconception have been linked to health issues in offspring, such as childhood cancers, low birth weight, preterm birth and pregnancy loss [13,69,70], only a few other studies have specifically connected them with an increased risk of birth defects [30]. Larger, more diverse population studies are needed to validate these findings and deepen our understanding of the underlying mechanisms.

This review consistently showed a strong link between paternal preconception smoking and a higher risk of CBDs, supporting a large body of existing research [71,72]. In men, smoking has been found to cause sperm DNA strand damage and increase chromosomal abnormalities, which can contribute to CBDs [73]. The review also noted that alcohol consumption was associated with an increased risk, especially for CHDs and anogenital distance. Previous research suggests that long-term alcohol abuse in men can lead to epigenetic changes that are passed down through generations. Abel [74] also indicated that paternal alcohol intake may contribute to fetal alcohol syndromes indirectly, through factors like couple conflict and maternal stress. Therefore, beyond its direct impact on sperm quality and gene expression, paternal substance use might influence CBD risk by affecting maternal health and wellbeing. These findings reinforce the importance of targeted public health strategies to encourage men to quit smoking and drinking before conception.

Our review emphasizes the importance of paternal environmental and occupational exposures—such as solvents, pesticides, heavy metals, and persistent organic pollutants—as factors contributing to both congenital malformations and reduced infant head circumference. The findings from the LIFE cohort and other studies on environmental exposures support a mechanism where paternal exposures can affect gamete quality or cause germline epigenetic changes. The smaller head circumference associated with paternal exposure to uranium, molybdenum, and PCBs is alarming and underscores the need for tighter regulation of hazardous substances in workplaces. Recent reviews focusing on the effects of environmental exposures on congenital anomalies have reached similar conclusions. Specifically, Boyd and colleagues [75] proposed that the interaction between genes and environmental factors could alter placental blood flow, thereby influencing the development of CHDs. Gaining a better understanding of these mechanisms would provide more comprehensive insights into how paternal preconception factors impact the risk of birth defects.

Although most medication classes showed no significant risk of congenital defects, a few notable exceptions emerged where metformin [42,43] and diazepam [55] were identified as potential risk factors for increased CBD risk. Paternal exposure to medications can affect fetal outcomes by influencing DNA mutations, sperm quality, and transferring medicinal components to the mother via seminal fluid [55]. However, our review found that some associations between medication exposure and health outcomes may be due to underlying physical health conditions rather than the medications themselves. Specifically, Meng and colleagues (2025) observed that paternal metformin use was associated with an increased risk of congenital malformation in the unadjusted analysis. Nevertheless, after accounting for factors such as type 2 diabetes mellitus and other potential confounders, the risk of malformation was no longer statistically significant. The authors concluded that confounding factors, such as advanced paternal age and paternal metabolic syndrome, might increase the risk of malformations. Therefore, more rigorous studies are needed to control for confounding variables and determine whether the medication or the underlying health condition is responsible for the observed effects.

While further research in this area would be valuable, challenges in studying paternal preconception health may arise from limitations such as the infrequency of birth defect cases in general medical practice and a lack of awareness about the importance of preconception counselling [76]. For example, most studies on medication exposure had large sample sizes; however, paternal exposure to specific types of drugs was smaller, and birth defects are an infrequent outcome. Thus, large international cohort studies with linked medication and neonatal outcomes are necessary to advance knowledge in this area. Nonetheless, the findings highlight the importance of a careful risk–benefit assessment when continuing paternal medications before conception, especially for chronic conditions.

### Head circumference

Compared to the strong findings for congenital defects, evidence for paternal exposures influencing head circumference remains mixed. Only six studies have examined paternal exposure and head circumference. One study indicated that higher paternal alcohol consumption was linked to microcephaly in offspring, although it did not find significant results when alcohol intake was measured as a continuous variable. This contradicts a previous study on periconceptional paternal alcohol consumption, which reported no significant changes in head circumference [77]. Additionally, paternal exposure to uranium and polychlorinated biphenyls (PCBs) was associated with a smaller head size. Uranium exposure typically occurs from mining activities and consuming contaminated groundwater [78], while PCBs are commonly found in certain foods and building materials [79,80]. Given the limited number of studies, further research on these exposures is essential.

### Strengths and limitations

The strengths of this review include a systematic, comprehensive search across multiple databases and the synthesis of various paternal exposure types. By examining different potential risk factors, this review provides a broad perspective on how paternal preconception health may influence CBDs and highlights areas for future research. It also highlights limitations and gaps in the paternal preconception health literature, suggesting directions for future investigation. One limitation is that most studies included were conducted with populations from Denmark, the US, and China, which could influence the generalisability of the findings. Another limitation is that some paternal preconception health factors were examined by only one or two studies (e.g., BMI, mental health), resulting in limited evidence and making it difficult to draw definitive conclusions. This highlights the clear discrepancy in the volume of preconception health research on women compared to men. Including more studies for each paternal risk factor would strengthen future scoping reviews. Further, for some variables known to affect maternal health (e.g., Zika virus) and CBD/microcephaly, no preconception studies were found for men. Many studies included in the scoping review controlled for potentially confounding variables. However, this varied across studies, with some controlling for baseline demographic characteristics (e.g., age, BMI) and others including broader health and lifestyle factors. Additionally, as this review focused on paternal health factors, it did not consider maternal or couple-related factors, which may also confound the associations between paternal exposures and birth outcomes. The prevalence of CBD may have been underestimated due to potential misclassification bias as most included studies evaluated CBDs among live births. Only a few studies included stillbirths, and no studies were identified that specifically included termination of pregnancy due to CBD. Lastly, in line with scoping review methodology, the quality of the included studies was not assessed. Future reviews could assess study quality, which is crucial for determining the overall significance of the evidence in informing recommendations.

## Conclusion

Our findings demonstrate that paternal health and exposures prior to conception—including chronic health conditions, substance use, environmental hazards, and specific medications—have quantifiable effects on offspring outcomes such as congenital anomalies and infant head growth. These results highlight the critical importance of expanding preconception care to actively involve fathers, with targeted strategies aimed at enhancing paternal health, eliminating tobacco and alcohol consumption, and mitigating occupational and environmental risks. Progress in this field necessitates large-scale, meticulously designed prospective studies employing biomarker-based exposure assessments to elucidate underlying mechanisms and inform evidence-based interventions. Augmenting the evidence regarding paternal contributions is vital for improving reproductive outcomes and promoting healthier developmental trajectories for the next generation

## Supporting information

S1 Table Search strategy for Medline. (DOCX)

S2 Table PRISMA-ScR Checklist. From: Tricco AC, Lillie E, Zarin W, O’Brien KK, Colquhoun H, Levac D, et al. PRISMA Extension for Scoping Reviews (PRISMAScR): Checklist and Explanation. Ann Intern Med. 2018;169:467–473. https://doi.org/10.7326/M18-0850.
(DOCX)
